# Mortality Risk Prediction in Patients With Antimelanoma Differentiation–Associated, Gene 5 Antibody–Positive, Dermatomyositis–Associated Interstitial Lung Disease: Algorithm Development and Validation

**DOI:** 10.2196/62836

**Published:** 2025-02-05

**Authors:** Hui Li, Ruyi Zou, Hongxia Xin, Ping He, Bin Xi, Yaqiong Tian, Qi Zhao, Xin Yan, Xiaohua Qiu, Yujuan Gao, Yin Liu, Min Cao, Bi Chen, Qian Han, Juan Chen, Guochun Wang, Hourong Cai

**Affiliations:** 1 Department of Respiratory and Critical Care Medicine Affiliated Drum Tower Hospital Nanjing University Medical School Nanjing China; 2 Department of Pulmonary and Critical Care Medicine Seventh Affiliated Hospital Sun Yatsen University Shenzhen China; 3 Department of Pulmonary and Critical Care Medicine General Hospital of Ningxia Medical University Yinchuan China; 4 Department of Respiratory and Critical Care Medicine Third People’s Hospital of Chengdu Chengdu China; 5 Department of Respiratory and Critical Care Medicine Affiliated Hospital of Xuzhou Medical University Xuzhou China; 6 National Center for Respiratory Medicine First Affiliated Hospital of Guangzhou Medical University Guangzhou China; 7 China-Japan Friendship Hospital Key Laboratory of Myositis (Beijing Key Laboratory for Immune Mediated Inflammatory Diseases) Department of Rheumatology Beijing China

**Keywords:** antimelanoma differentiation–associated gene 5 antibody, dermatomyositis, interstitial lung disease, 3-month mortality, machine learning, ML, tool, web based, mortality, idiopathic inflammatory myopathy, myopathy, lung disease, melanoma, imaging, clinical outcome

## Abstract

**Background:**

Patients with antimelanoma differentiation–associated gene 5 antibody–positive dermatomyositis–associated interstitial lung disease (anti-MDA5+DM-ILD) are susceptible to rapidly progressive interstitial lung disease (RP-ILD) and have a high risk of mortality. There is an urgent need for a reliable prediction model, accessible via an easy-to-use web-based tool, to evaluate the risk of death.

**Objective:**

This study aimed to develop and validate a risk prediction model of 3-month mortality using machine learning (ML) in a large multicenter cohort of patients with anti-MDA5+DM-ILD in China.

**Methods:**

In total, 609 consecutive patients with anti-MDA5+DM-ILD were retrospectively enrolled from 6 hospitals across China. Patient demographics and laboratory and clinical parameters were collected on admission. The primary endpoint was 3-month mortality due to all causes. Six ML algorithms (Extreme Gradient Boosting [XGBoost], logistic regression (LR), Light Gradient Boosting Machine [LightGBM], random forest [RF], support vector machine [SVM], and k-nearest neighbor [KNN]) were applied to construct and evaluate the model.

**Results:**

After applying inclusion and exclusion criteria, 509 (83.6%) of the 609 patients were included in our study, divided into a training cohort (n=203, 39.9%), an internal validation cohort (n=51, 10%), and 2 external validation cohorts (n=92, 18.1%, and n=163, 32%). ML identified 8 important variables as critical for model construction: RP-ILD, erythrocyte sedimentation rate (ESR), serum albumin (ALB) level, age, C-reactive protein (CRP) level, aspartate aminotransferase (AST) level, lactate dehydrogenase (LDH) level, and the neutrophil-to-lymphocyte ratio (NLR). LR was chosen as the best algorithm for model construction, and the model demonstrated excellent performance, with an area under the receiver operating characteristic (ROC) curve (AUC) of 0.866, a sensitivity of 84.8%, and a specificity of 84.4% on the validation data set and an AUC of 0.90, a sensitivity of 85.0%, and a specificity of 83.9% on the training data set. Calibration curves and decision curve analysis (DCA) confirmed the model’s accuracy and clinical applicability. Moreover, the model showed strong predictive performance in the external validation cohorts (cohort 1: AUC=0.836, 95% CI 0.754-0.916; cohort 2: AUC=0.915, 95% CI 0.871-0.959), indicating good generalizability. This model was integrated into a web-based tool to predict the 3-month mortality for patients with anti-MDA5+DM-ILD.

**Conclusions:**

We successfully developed a robust clinical prediction model and an accompanying web tool to estimate the 3-month mortality risk for patients with anti-MDA5+DM-ILD.

## Introduction

Idiopathic inflammatory myopathies (IIMs) encompass a heterogenous group of autoimmune diseases characterized by muscle weakness and inflammation. Dermatomyositis (DM), which predominantly affects the skin and muscles, is a prevalent subtype within the IIM spectrum. Interstitial lung disease (ILD) is a common comorbidity in DM, with an incidence varying widely from 5% to 65% [1]. The clinical presentations, treatment responses, and outcomes of IIMs are highly variable and correlate with the presence of specific myositis-specific antibodies (MSAs). One such antibody, the antimelanoma differentiation–associated gene 5 (MDA5) antibody, targets the MDA5 protein, a member of the retinoic acid inducible gene I (RIG-I) receptor family, which is crucial for the detection of viral RNA and the activation of the interferon pathway in response to viral infections [2]. Approximately 1%-30% of patients with DM test positive for anti-MDA5 antibodies [3]. A recent surge in MDA5+DM cases has been observed in England in the wake of the COVID-19 pandemic, with an associated mortality rate of 32% for antimelanoma differentiation–associated gene 5 antibody–positive dermatomyositis–associated interstitial lung disease (anti-MDA5+DM-ILD) [4]. Patients with anti-MDA5 autoantibody often exhibit minimal muscle involvement. Initially, anti-MDA5 autoantibody was identified in a cohort of East Asian patients with clinically amyopathic myositis (81%) and rapidly progressive ILD (74%) [5], with >90% of rapidly progressive interstitial lung disease (RP-ILD) cases and 84% mortality occurring within the first 6 months of disease onset [6]. Notably, the first 3 months following diagnosis are particularly critical, with 46% of the deaths occurring in this period [6]. However, recent studies have revealed that early and aggressive intervention can significantly improve the 6-month survival rate of patients with anti-MDA5+DM-ILD from 33% to 89% [7-9]. Thus, there is an urgent need to accurately identify these high-risk patients at an early stage of the disease.

In recent years, various mortality risk models incorporating independent predictors have been explored to predict outcomes in patients with anti-MDA5+DM-ILD. Lian et al [10] developed the FLAIR score, which includes 5 key indicators: ferritin levels, lactate dehydrogenase (LDH) levels, the anti-MDA5 antibody grade, the high-resolution computed tomography (HRCT) imaging score, and RP-ILD [10]. However, the FLAIR score was designed to predict mortality in patients with amyopathic DM. Some independent prognostic factors, such as Krebs von den Lungen-6 (KL-6), interleukin (IL)-6, IL-18, and soluble CD206, are challenging to access in clinical practice [11], and HRCT imaging scores are subject to considerable interobserver variability [10]. Most existing prediction models rely on logistic or Cox regression analysis, with limited external validation and only fair overall performance. Recent evidence suggests that machine learning (ML) algorithms, which can handle numerous multidimensional variables with nonlinear relationships, may offer superior predictive accuracy for clinical outcomes [12]. To date, ML approaches for predicting mortality risk and prognosis in patients with anti-MDA5+DM-ILD have been infrequent, and there remains a lack of a concise, practical tool for directly calculating mortality risk in these patients.

Considering the limited sample sizes and inadequate establishment and validation of practical quantitative methods in previous studies, we identified the 3-month mortality risk, established a prediction model, and validated the model using real-world data from multicenter studies in China involving patients with anti-MDA5+DM-ILD. The aim was to create a reliable and easily implemented tool for clinical use.

## Methods

### Ethical Considerations

The Medical Ethics Committee of the Drum Tower Hospital Affiliated to Nanjing University Medical School approved the study (2020-050-01). The patients included in this study were derived from a secondary analysis of the previously confirmed study. The ethics committee determined that a second round of approval was not required.

### Patients and Study Design

From January 2017 to December 2022, our study screened patients with anti-MDA5+DM-ILD across participating institutions. Inclusion criteria were patients with a newly observed ILD, detectable serum anti-MDA5, and DM. The diagnostic criteria for DM included Bohan and Peter criteria and were retrospectively reconfirmed according to the 2017 European League Against Rheumatism (EULAR)/American College of Rheumatology IIM classification criteria or the 2018 European Neuromuscular Centre (ENMC) DM criteria by experienced rheumatologists and pulmonary clinicians [13-15]. Exclusion criteria were as follows: (1) DM-ILD with other positive MSAs; (2) other connective tissue diseases (CTDs); (3) evidence of pulmonary infections at initial admission, as diagnosed clinically and by identifying pathogens; and (4) incomplete data or lost to follow-up.

Following application of these criteria, 509 (83.6%) eligible cases from an initial 609 patients were identified. Patients from the Nanjing Drum Tower Hospital (NJDTH; n=254, 49.9%) were randomly allocated to a training data set (n=203, 79.9%) for model development and internal validation using k-fold cross-validation and a testing set (n=51, 20.1%) for further model validation. External validation was conducted using cohorts from 4 additional institutes, referred to as external cohort 1 (n=92, 18.1%) and external cohort 2 (n=163, 32%).

### Clinical Data Collection and Reference Standards

All clinical and laboratory data were extracted from electronic medical records (EMRs) at the beginning of the patients’ admission. Demographic characteristics, including age and gender, and clinical signs, such as muscle weakness, the Raynaud phenomenon, mechanic’s hands, Gottron sign/papules, heliotrope rash, fever, and arthralgia/arthritis, were documented and compared. Serum levels of muscle enzymes, such as creatine kinase (CK), alanine aminotransferase (ALT), aspartate aminotransferase (AST), and lactate dehydrogenase (LDH), were recorded. Serum antibody abnormalities, including Ro/SSA-52kDa antibody (Ro52) and MDA5 (autoimmune inflammatory myopathies 16 Ag-(IgG); EUROLINE), were analyzed. Disease severity evaluations and inflammatory mediators, such as C-reactive protein (CRP), the erythrocyte sedimentation rate (ESR), the white blood cell (WBC) count, the lymphocyte count, the neutrophil-to-lymphocyte ratio (NLR), and ferritin levels, were recorded and compared. In this study, all these variables were meticulously recorded during hospitalization, with a low incidence of missing data of less than 2%. Missing data were processed with the k-nearest neighbor (KNN) algorithm.

ILD has been defined by the American Thoracic Society/European Respiratory Society [16]. RP-ILD is characterized by the acute and progressive deterioration of ILD, manifesting as worsening dyspnea and interstitial changes on chest HRCT, necessitating hospitalization due to disease-related acute respiratory failure within 3 months of the onset of respiratory symptoms, after exclusion of other potential etiologies (eg, pulmonary infection, heart failure, embolism) [17,18]. The duration of the disease is marked from the initial occurrence of pulmonary, muscle, joint, or skin symptoms/signs.

The primary outcome was the risk of mortality at 3 months. Secondary outcomes included the 1-month mortality risk and the 6-month mortality risk.

### Screening of Important Features for Model Construction

We used the BorutaShap algorithm for feature selection, which integrates Boruta and Shapley Additive Explanations (SHAP) algorithms to pinpoint the most influential features within a data set [19]. Consequently, the BorutaShap algorithm was used to sift through 25 parameters, including demographics, clinical features, and laboratory parameters, from the EMR system and identify the most pertinent features for model construction. In this study, only features categorized as “accepted” by the BorutaShap algorithm were incorporated into model development. Features labeled “tentative” or “rejected” were omitted.

### Establishment and Validation of Prediction Models

Six ML algorithms were deployed to build prediction models: Extreme Gradient Boosting (XGBoost), logistic regression (LR), Light Gradient Boosting Machine (LightGBM), random forest (RF), support vector machine (SVM), and KNN. To guarantee robustness and reproducibility, all models were provided with identical input features. A thorough search of the hyperparameter space was conducted using both grid and random searches to optimize each model’s performance. The reliability of these models was appraised using various evaluation metrics, including the area under the receiver operating characteristic (ROC) curve (AUC), sensitivity, specificity, the positive predictive value (PPV), the negative predictive value (NPV), accuracy, and the *F*_1_-score. Moreover, 10-fold cross-validations were implemented in the derivation cohort to validate the models.

The optimal model was elucidated using the SHAP methodology, which delineates the importance and contribution of each feature to the model. The model results were interpreted by qualifying the impact of each feature on the predictions. Ultimately, the optimal prediction model was integrated into a user-friendly web-based tool.

### Statistical Analysis

Descriptive statistics were applied to summarize continuous variables as mean (SD) values and categorical variables as frequencies and percentage. The Fisher exact test was performed for categorical variables, while the Mann-Whitney *U* test was used for continuous variables to assess differences between the survival and mortality groups within 3 months. Statistical significance was set at *P*<.05. All statistical analyses were performed using SPSS Statistics version 26.0 (IBM Corporation), R Studio version 3.6.3 (R Foundation for Statistical Computing) and Python version 3.7.

## Results

### Patients’ Basic Clinical Characteristics

**Figure 1:**
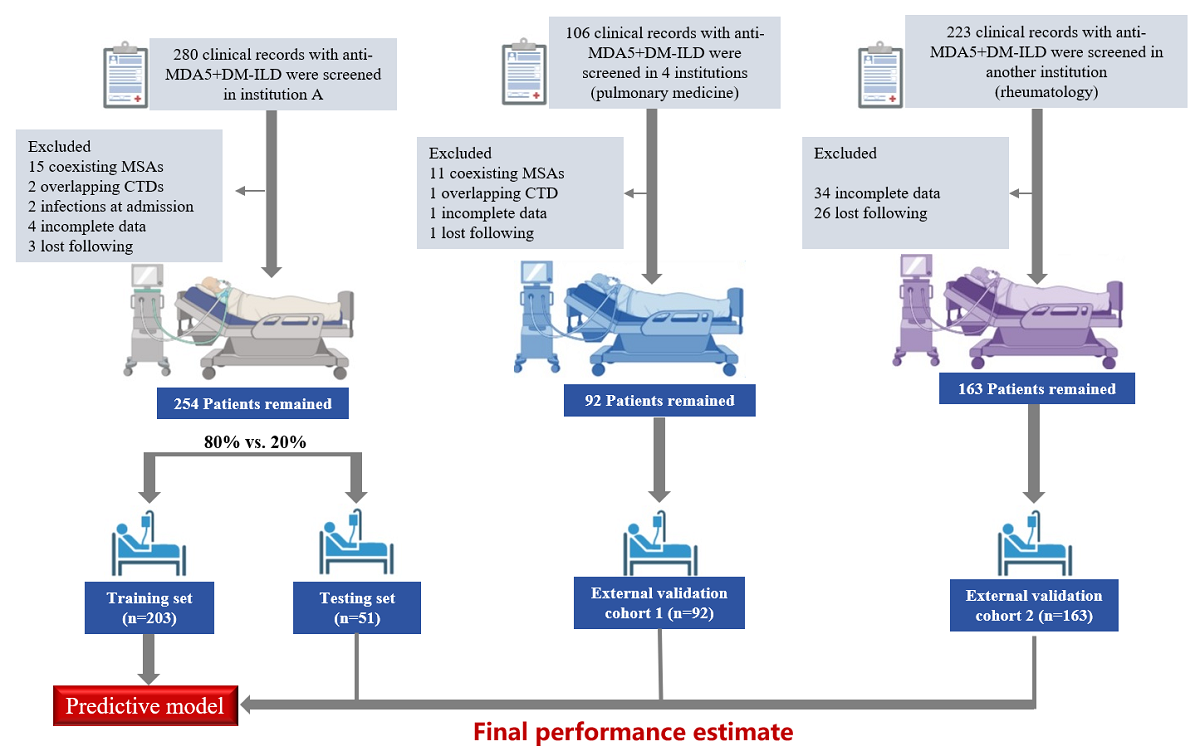
Flowchart of patient selection and development of a predictive model. Anti-MDA5+DM-ILD: melanoma differentiation–associated gene 5 antibody–positive dermatomyositis–associated interstitial lung disease; CTD: connective tissue disease.

The study flowchart is shown in Figure 1. The NJDTH cohort had 280 (55%) patients with anti-MDA5+DM-ILD, and after exclusion, the final count was 254 (49.9%) patients. These patients were followed for a median of 6 months, divided into a training cohort of 203 (79.9%) patients and an internal testing cohort of 51 (20.1%) patients. The external validation cohort was further refined to 92 (18.1%) patients in cohort 1 and 163 (32%) patients in external cohort 2 (Figure 1). The entire patient group was analyzed for all-cause mortality. A comparative baseline analysis of 31 clinical variables, encompassing demographics, clinical severity, inflammatory indicators, serum antibody levels, is presented in Table 1. The study evaluated statistically significant differences in these variables among the groups. Of 254 valid samples, 159 (62.6%) were in the 3-month survival group and 95 (37.4%) in the 3-month death group in the NJDTH cohort. In terms of mortality, 108 (42.5%) patients died during the investigation period, with 71 (27.9%) within 30 days, 95 (37.4%) within 90 days, and 101 (39.78%) within 180 days. The 3-month mortality was up to 37.4%, suggesting the majority of deaths occurred within the first 3 months. Patients who died within 3 months showed more severe injury, as indicated by higher levels of inflammatory markers, such as CRP, ESR, LDH, WBC, ferritin, and NLR, and lower levels of lymphocytes compared to patients who survived beyond 3 months. RP-ILD characteristics were observed in 78 (82.1%) of 95 patients who died within 3 months in contrast to only 30 (18.9%) of 159 survivors. The mean age of patients who died within 3 months was 56.68 (SD 9.97) years, which was higher than that of patients who survived for 3 months (mean 51.50, SD 10.68 years). There were no statistically significant differences in patients’ characteristics between the training and testing sets. Other severity indices were comparable between the training and testing sets (Table 2).

**Table 1:** Comparison of baseline clinical parameters and all-cause death data of patients from the NJDTH^a^ cohort.

Variables		Total NJDTH cohort (N=254)	3-month survival (n=159)	3-month death (n=95)	*P* value
Age (years), mean (SD)		53.44 (10.72)	51.50 (10.68)	56.68 (9.97)	<.001
**Gender, n (%)**					
	Male	77 (30.3)	47 (29.6)	30 (31.6)	.735
	Female	177 (69.7)	112 (70.4)	65 (68.4)	N/A^b^
Smoking, n (%)		33 (13.0)	17 (10.7)	16 (16.8)	.158
**Clinical features**					
	Survival time (months)	13.03	28.70	0.50	<.001
	Duration (months)	2.00	2.00	1.00	<.001
	RP-ILD^c^, n (%)	108 (42.5)	30 (18.9)	78 (82.1)	<.001
	Muscle weakness, n (%)	63 (24.8)	46 (28.9)	17 (17.9)	.049
	Raynaud phenomenon, n (%)	13 (5.1)	9 (5.7)	4 (4.2)	.612
	Mechanic’s hands, n (%)	74 (29.1)	42 (26.4)	32 (33.7)	.217
	Gottron sign/papules, n (%)	167 (65.6)	110 (69.2)	57 (60.0)	.136
	Heliotrope rash, n (%)	114 (45.1)	78 (49.4)	36 (37.9)	.076
	RF^d^, n (%)	19 (7.5)	15 (9.4)	4 (4.2)	.126
	ANA^e^, n (%)	103 (40.6)	69 (43.4)	34 (35.8)	.232
	Fever, n (%)	132 (52.0)	65 (40.9)	67 (70.5)	<.001
	Arthralgia/arthritis, n (%)	87 (34.3)	68 (42.8)	19 (20.0)	<.001
**Laboratory parameters**					
	Ro52^f^, n (%)	177 (69.7)	105 (66.0)	72 (75.8)	.102
	MDA5^g^ grade +, n (%)	53 (20.9)	36 (22.6)	17 (17.9)	.665
	MDA5 grade ++, n (%)	65 (25.6)	40 (25.2)	25 (26.3)	N/A
	MDA5 grade +++, n (%)	136 (53.5)	83 (52.2)	53 (55.8)	N/A
	ALB^h^ (g/L)	33.57	34.70	31.66	<.001
	ALT^i^ (U/L)	51.70	44.10	60.80	.021
	AST^j^ (U/L)	43.30	37.60	56.10	<.001
	CK^k^ (U/L)	33.00	31.00	41.00	.188
	LDH^l^ (U/L)	349.00	304.00	439.00	<.001
	CRP^m^ (mg/L)	6.00	4.70	15.40	<.001
	ESR^n^ (mm/hour)	39.00	33.00	46.00	<.001
	WBC^o^ (×10^9^)	6.00	5.50	6.50	.003
	Lymphocytes (×10^9^)	0.70	0.80	0.70	.011
	NLR^p^	5.50	5.00	8.57	<.001
	PaO_2_/FiO_2_^q^	248.00	328.00	185.00	<.001
	Ferritin (ng/mL)	1063.5	698.30	1650.00	<.001

^a^NJDTH: Nanjing Drum Tower Hospital.

^b^N/A: not applicable.

^c^RP-ILD: rapidly progressive interstitial lung disease.

^d^RF: rheumatoid factor.

^e^ANA: antinuclear antibody.

^f^Ro52: Ro/SSA-52kDa antibody.

^g^MDA5: melanoma differentiation–associated gene 5.

^h^ALB: albumin.

^i^ALT: alanine aminotransferase.

^j^AST: aspartate aminotransferase.

^k^CK: creatine kinase.

^l^LDH: lactate dehydrogenase.

^m^CRP: C-reactive protein.

^n^ESR: erythrocyte sedimentation rate.

^o^WBC: white blood cell.

^p^NLR: neutrophil-to-lymphocyte ratio.

^q^PaO_2_/FiO_2_: ratio of partial pressure of oxygen in arterial blood to fraction of inspired oxygen.

**Table 2:** Comparison of baseline parameters among training, testing, and validation cohorts.

Variables			Total participants (N=509)		Training cohort (n=203)		Testing cohort (n=51)		External validation cohort 1 (n=92)		External validation cohort 2 (n=163)
Age (years), mean (SD)			53.2 (11.2)		52.9 (11.5)		54.0 (10.2)		52.6 (10.4)		49.3 (11.2)
**Gender, n (%)**											
	Male	170 (33.4)		59 (29.1)		18 (35.3)		40 (43.5)		53 (32.5)	
	Female	339 (66.6)		144 (70.9)		33 (64.7)		52 (56.5)		110 (67.5)	
Smoking, n (%)			87 (17.1)		25 (12.3)		8 (15.7)		17 (18.5)		37 (22.7)
**Clinical features**											
	Duration (months)	2.00		2.00		1.00		2.00		3.00	
	RP-ILD^a^, n (%)	198 (38.9)		88 (43.4)		20 (39.2)		42 (45.7)		48 (29.4)	
	Heliotrope rash, n (%)	267 (52.6)		90 (44.6)		24 (47.1)		36 (39.1)		117 (71.8)	
	Gottron sign/papules, n (%)	337 (66.2)		136 (67.0)		31 (60.8)		35 (38.0)		135 (82.8)	
	Raynaud phenomenon, n (%)	29 (5.7)		13 (6.4)		0		3 (3.3)		13 (8.0)	
	Mechanic’s hands, n (%)	194 (38.1)		61 (30.1)		13 (25.5)		33 (35.9)		87 (53.4)	
	Muscle weakness, n (%)	165 (32.4)		50 (24.6)		13 (25.5)		26 (28.3)		76 (46.6)	
	Arthralgia/arthritis, n (%)	183 (36.0)		71 (35.0)		16 (31.4)		39 (42.4)		57 (35.0)	
	Fever, n (%)	224 (44.0)		108 (53.2)		24 (47.1)		31 (33.7)		61 (37.4)	
	Skin ulcer, n (%)	90 (19.2)		24 (11.8)		11 (21.6)		7 (13.5)		48 (29.5)	
**Laboratory parameters**											
	Ro52^b^, n (%)	328 (65.1)		140 (69.0)		37 (72.6)		62 (67.4)		89 (56.3)	
	RF^c^, n (%)	49 (9.8)		14 (6.9)		5 (9.8)		10 (10.9)		20 (13.1)	
	ANA^d^, n (%)	232 (46.2)		80 (39.4)		23 (45.1)		43 (47.3)		86 (54.8)	
	ALB^e^ (g/L)	33.40		33.30		34.10		30.50		34.70	
	ALT^f^ (IU/L)	47.70		53.90		47.70		42.40		44.00	
	AST^g^ (IU/L)	45.50		41.90		50.90		61.00		40.00	
	CK^h^ (IU/L)	40.90		33.00		30.00		87.00		41.00	
	LDH^i^ (IU/L)	335.00		351.00		349.00		392.00		297.00	
	CRP^j^ (mg/L)	5.20		6.00		6.60		6.83		4.01	
	ESR^k^ (mm/hour)	34.00		38.00		42.00		39.00		25.00	
	WBC^l^ (×10^9^)	5.91		5.90		6.20		5.90		5.91	
	Lymphocytes (×10^9^)	0.71		0.70		0.81		0.69		0.74	
	NLR^m^	33.40		6.00		5.25		6.94		4.98	
	PaO_2_/FiO_2_^n^	309.00		254.00		224.00		248.00		391.91	
	Ferritin (ng/mL)	804.20		1048.60		1164.30		1351.00		475.10	
**Primary outcome** **, n (%)**											
	Death at 3 months	178 (35.0)		75 (37.0)		20 (39.2)		46 (50.0)		37 (22.7)	
**Secondary outcomes** **, n (%)**											
	Death at 1 month	129 (25.3)		55 (27.1)		16 (31.4)		26 (28.3)		32 (19.6)	
	Death at 6 months	191 (37.5)		80 (39.4)		21 (41.2)		50 (54.4)		40 (24.5)	
	All deaths	202 (39.7)		87 (42.9)		21 (41.2)		52 (56.5)		42 (25.8)	

^a^RP-ILD: rapidly progressive interstitial lung disease.

^b^Ro52: Ro/SSA-52kDa antibody.

^c^RF: rheumatoid factor.

^d^ANA: antinuclear antibody.

^e^ALB: albumin.

^f^ALT: alanine aminotransferase.

^g^AST: aspartate aminotransferase.

^h^CK: creatine kinase.

^i^LDH: lactate dehydrogenase.

^j^CRP: C-reactive protein.

^k^ESR: erythrocyte sedimentation rate.

^l^WBC: white blood cell.

^m^NLR: neutrophil-to-lymphocyte ratio.

^n^PaO_2_/FiO_2_: ratio of partial pressure of oxygen in arterial blood to fraction of inspired oxygen.

To account for varying severities of with anti-MDA5+DM-ILD patients, we recruited external cohort 1 mainly from the pulmonary medicine department and external cohort 2 from the rheumatology department. Patients in external cohort 1 tended to have severe illness than those in external cohort 2, as patients with respiratory failure were more likely to be admitted to the pulmonary medicine department. Analysis on a diverse range of severities of patients enhanced our model’s practicality and robustness.

### Screening of Characteristic Factors for Risk of 3-Month Mortality

**Figure 2:**
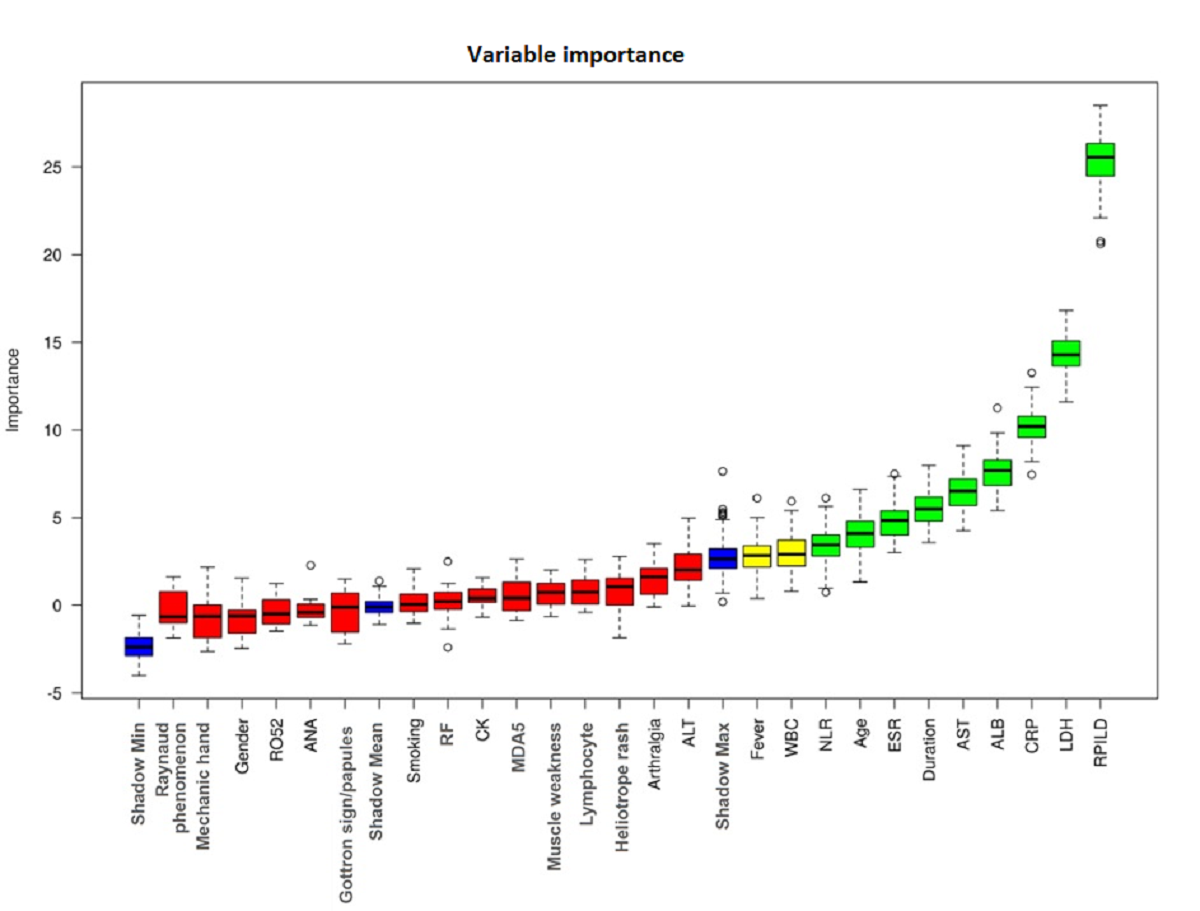
By using the BorutaShap algorithm, selected parameters or patients’ characteristics were calculated for the importance of evaluation. Parameters with green color were categorized as “accepted” for the next step of analysis ALB: albumin; ALT: alanine aminotransferase; ANA: antinuclear antibody; AST: aspartate aminotransferase; CK: creatine kinase; CRP: C-reactive protein; ESR: erythrocyte sedimentation rate; LDH: lactate dehydrogenase; MDA5: melanoma differentiation–associated gene 5; NLR: neutrophil-to-lymphocyte ratio; RF: rheumatoid factor; RP-ILD: rapidly progressive interstitial lung disease; SHAP: Shapley Additive Explanations; WBC: white blood cell.

In total, 9 parameters (ie, RP-ILD, age, LDH, albumin [ALB], CRP, ESR, AST, NLR, and duration of disease) were selected for analysis using the BorutaShap algorithm (Figure 2). However, due to the overlap between disease duration and RP-ILD, RP-ILD was left for model development to balance model complexity and generalizability. Hence, 8 parameters were selected for further analysis.

### Comprehensive Analysis of the Prediction Model and Selection of the Optimal Model

**Figure 3:**
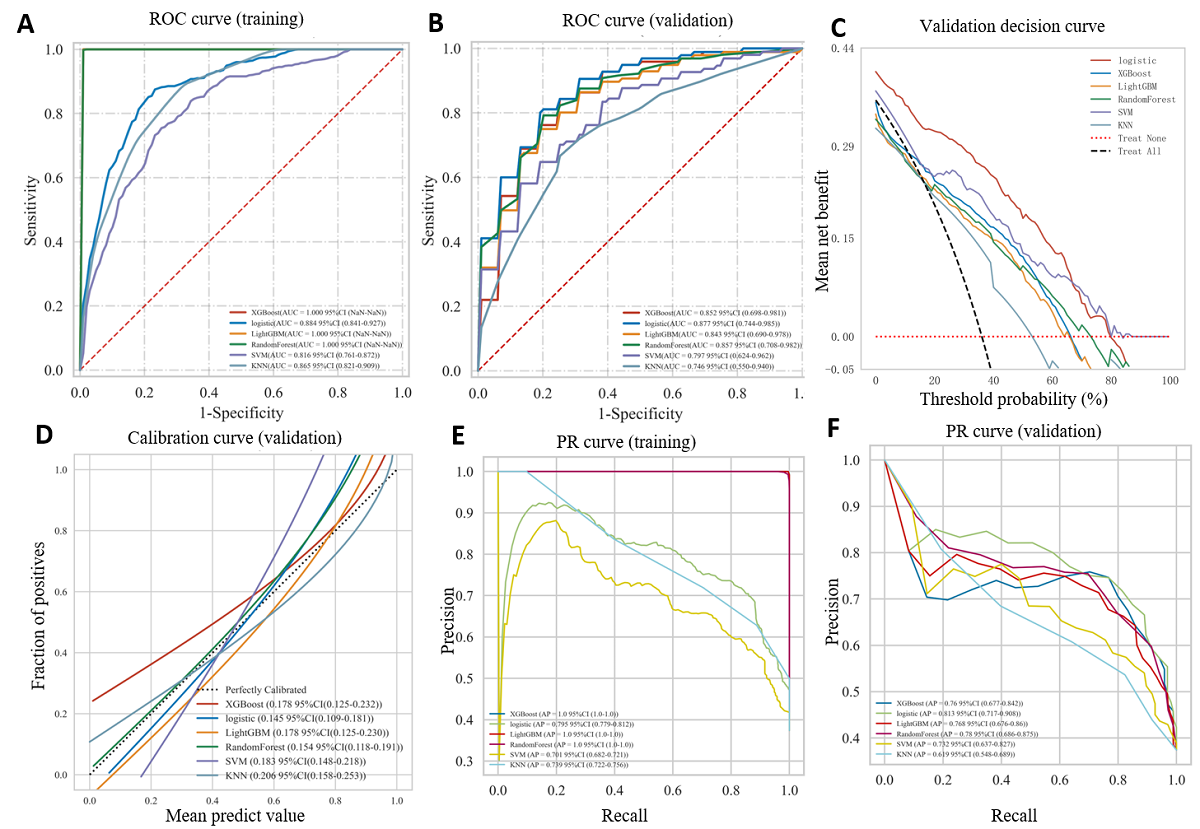
Comparison of prediction models for performance using 6 ML algorithms. ROC results for predicting 3-month mortality obtained by using different models for the (A) training cohort and (B) validation cohort. (C) DCA, (D), calibration curves, and (E, F) PR curves were used to verify which model was preferable. DCA: decision curve analysis; KNN: k-nearest neighbor; LightGBM: Light Gradient Boosting Machine; LR: logistic regression; ML: machine learning; PR: precision-recall; RF: rheumatoid factor; ROC: receiver operating characteristic; SVM: support vector machine; XGBoost: Extreme Gradient Boosting.

To identify the best prediction model for the 3-month mortality risk in patients with anti-MDA5+DM-ILD, all models were evaluated using identical input features and assessed via AUC values, decision curve analysis (DCA), calibration curves, and precision-recall (PR) curves. XGBoost, LightGBM, and RF algorithms showed the highest AUCs in the training data set, but LR demonstrated the highest AUC of 0.877 (95% CI 0.744-0.985) in the validation data set (Figure 3A,B). Tables S1 and S2 in Multimedia Appendix 1 provide more details, indicating potential overfitting in the XGBoost, LightGBM, and RF algorithms. Furthermore, the LR model achieved a significantly lower Brier score of 0.145 compared to other models. The DCA curves in Figure 3C and the calibration plots in Figure 3D demonstrate that all 6 models provided a net clinical benefit over the full treat-all or treat-none strategies, with the LR model outperforming the other 5 models in the validation data set. Additionally, the LR model obtained the highest average precision (AP) value of 0.813 on the validation data set (Figure 3E,F). Based on these findings, the LR model was selected as the final model due to its superior performance.

**Figure 4:**
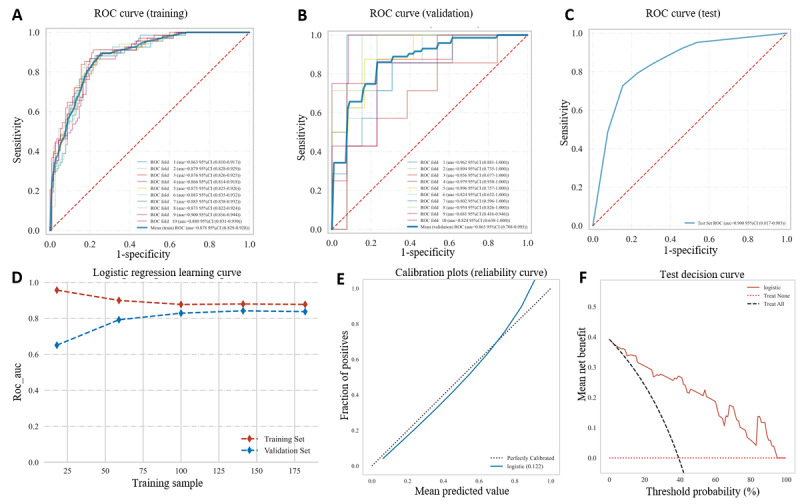
Performance of the LR model. (A) Training data set ROC and AUC and (B) internal cross-validation set ROC and AUC. The red dashed line represents the training data set, and the blue dashed line represents the validation data set. Values are expressed as averages (95% CI). Solid lines of different colors represent 10 different results. (C) Training data set ROC and AUC. Test results for patients. (D) Learning curve in training and validation data set, (E) calibration curves, and (F) DCA. AUC: area under the receiver operating characteristic curve; DCA: decision curve analysis; LR: logistic regression; ROC: receiver operating characteristic.

Ultimately, we selected RP-ILD, age, LDH, AST, ALB, NLR, CRP, and ESR as input features for the LR model. As shown in Figure 4 and Table S3 in Multimedia Appendix 1, the LR model demonstrated optimal performance with an AUC, sensitivity, specificity, and accuracy of 0.86, 84.8%, 84.4%, and 79.3%, respectively, on the validation data set and 0.90, 85.0%, 83.9%, and 84.3%, respectively, on the training data set (Figure 4A-D). The calibration of the LR model indicated strong concordance between the predicted probabilities and the actual observed 3-month deaths using Brier scores (Figure 4E). DCA revealed that the model provided greater net benefits over both treat-all and treat-none strategies (Figure 4F).

### SHAP Model Interpretation and Individual Analysis

**Figure 5:**
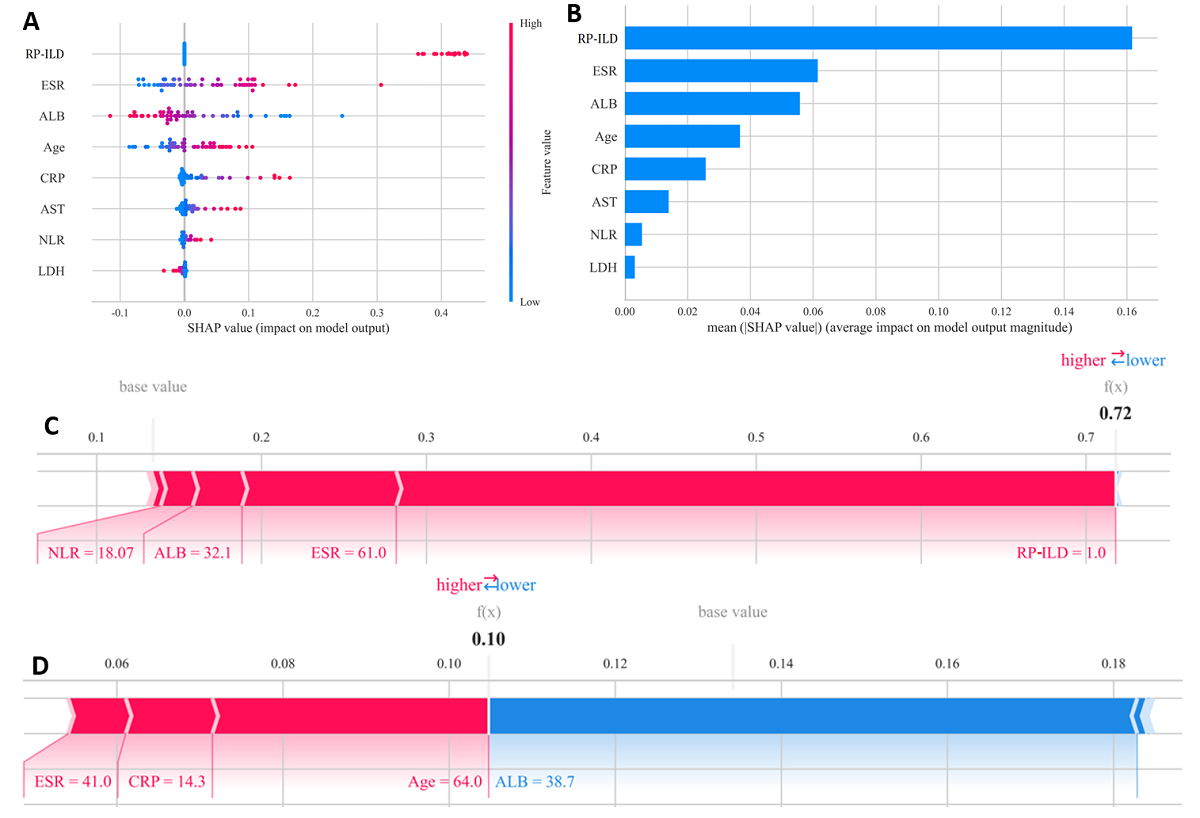
SHAP interpretation of the model. (A) Attributes of characteristics in the SHAP chart, (B) mean SHAP value of 8 important variables, (C, D) 2 clinical cases illustrating the usage of a weighing model. ALB: albumin; AST: aspartate aminotransferase; CRP: C-reactive protein; ESR: erythrocyte sedimentation rate; LDH: lactate dehydrogenase; NLR: neutrophil-to-lymphocyte ratio; RP-ILD: rapidly progressive interstitial lung disease; SHAP: Shapley Additive Explanations.

To provide a clear explanation of the selected variables, we used SHAP to illustrate how these variables predict the 3-month mortality within the model. Figure 5A displays the 8 most influential features (RP-ILD, ESR, ALB, age, CRP, AST, NLR, and LDH) in our model. Each feature’s importance line is represented by colored dots, where red dots indicate high-risk values and blue dots signify low-risk values. Figure 5B ranks these 8 risk factors based on their average absolute SHAP values, with the x axis representing SHAP values that denote the prediction model’s importance. Additionally, in this model, the probability threshold for the 3-month mortality risk was established at 0.40473. We then provided 2 exemplary cases to illustrate the model’s interpretability. In one instance, a patient presenting with RP-ILD exhibited a notably elevated SHAP-predicted mortality risk of 0.718 (Figure 5C), and unfortunately, this individual succumbed to the disease within a 3-month period postonset. In contrast, a 64-year-old participant demonstrated a substantially lower SHAP-predicted mortality risk of 0.105 (Figure 5D) and remained alive, underscoring the potential utility of the web-based prognostic tool in clinical practice.

### Model External Validation and Establishment of a Web-Based Calculator

**Figure 6:**
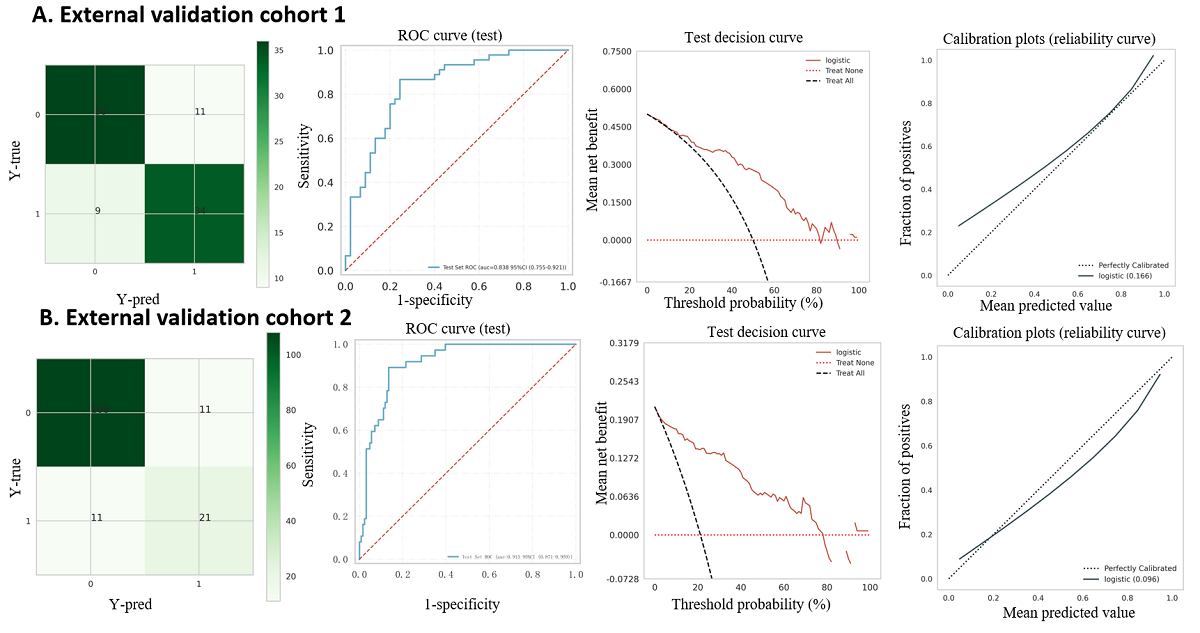
AUC and ROC of the LR model in the 2 external validation cohorts. AUC: area under the receiver operating characteristic curve; LR: logistic regression; ROC: receiver operating characteristic.

The AUC of our proposed model on external cohort 1 from 4 hospitals in China was 0.836 (Figure 6). The sensitivity, specificity, and accuracy for this cohort were 0.87, 0.739, and 0.783, respectively. For external cohort 2, the AUC, sensitivity, specificity, and accuracy were 0.915, 0.892, 0.865, and 0.847, respectively, indicating the model’s robust extrapolation capabilities. In addition, secondary outcomes (1-month and 6-month mortality risks) were evaluated in the 2 external validation cohorts. The model’s superior discrimination performance, using the AUC, sensitivity, specificity, precision, accuracy, and the *F*_1_-score, is detailed in Tables S4 and S5 in Multimedia Appendix 1, Figure S1 in Multimedia Appendix 2, and Figure S2 in Multimedia Appendix 3.

To enhance generalizability and facilitate clinical application, the optimal ML model was adapted to create a web-based calculator [20]. The calculator serves as a practical clinical tool for physicians specializing in respiratory medicine, rheumatology, and critical care medicine. It is particularly useful in the assessment of patients presenting with RP-ILD attributed to anti-MDA5+DM-ILD. A score exceeding 40.47% indicates a heightened risk of mortality, warranting consideration for aggressive management strategies.

## Discussion

### Principal Findings

To the best of our knowledge, this is the largest retrospective multicenter study to investigate the risk factors for 3-month mortality and to explore 6 ML models for comprehensive predictive analyses on anti-MDA5+DM-ILD cohorts in China. We identified a set of predictive risk factors and constructed a prediction model for patients with anti-MDA5+DM-ILD using ML algorithms in conjunction with clinical and laboratory data easily extracted from the EMR system. In this study, LR, using routinely collected data from real-world clinical practice, demonstrated superior performance across 8 predictive measures. The optimal model exhibited favorable discrimination and calibration, while also emphasizing explainability to enhance trust and transparency. Furthermore, variable importance was investigated at both individual and population levels. Additionally, external validation confirmed the superior predictive performance of this optimal model. To facilitate clinical application, a web-based tool was developed for health care professionals to use the proposed prediction model.

Previous studies have consistently reported high mortality rates exceeding 60% among patients with anti-MDA5+DM-ILD, particularly in East Asian populations, such as China [21]. The majority of deaths occurred within the first 3 months [22]. It is widely recognized that an excessive inflammatory response is associated with a heightened risk of RP-ILD and all-cause mortality [23]. Hyperinflammation plays a crucial role in the development and progression of anti-MDA5+DM-ILD. Uncontrolled hyperinflammation and persistent immune activation contribute to severe lung injury and RP-ILD, which may resemble the phenomenon known as “cytokine storm” observed in COVID-19 [24]. Previous studies have indicated that poor outcomes in patients with DM are correlated with certain factors, including advanced age and elevated levels of serum ferritin, LDH, NLR, and CRP [21]. Consistent with these findings, we established a prediction model incorporating 8 key variables of significant predictive importance: RP-ILD, ESR, ALB, age, CRP, AST, NLR, and LDH. Most of these variables are inflammatory markers that can reflect the hyperinflammatory state and were easy to obtain and observe in previous studies on patients with DM in a clinical setting. Moreover, classical statistical analysis has identified risk factors in these patient populations, including RP-ILD and age [21,25,26]. In line with previous findings, RP-ILD was found to be the most important feature according to the SHAP value and contributed to this prediction model. Age was ranked fourth in terms of its significance as a parameter associated with worsened outcomes. Therefore, this prediction model demonstrated superior performance, yielding AUCs of 0.90 in the internal cohorts and 0.832-0.919 in external cohorts.

There are currently no existing ML-based prediction models for 3-month mortality in individuals with anti-MDA5+DM-ILD, making it impossible to make a direct comparison with prior work. Previous studies have used logistic or Cox proportional hazard models as alternatives [10,27-29]. For instance, a nomogram model incorporating the duration of first-time symptoms, the presence of fever, pleural effusion (PE), total CT scores, and AST has been recommended as a prognostic predictor for anti-MDA5+DM-ILD outcomes [29]. Another model known as the “FLAIR score,” which combines ferritin, LDH, the semiquantitative anti-MDA5 grade, the HRCT imaging score, and RP-ILD/non-RP-ILD classification, was proposed to predict mortality in amyopathic DM-related ILD based on a large-scale Chinese single-center cohort study [10]. In contrast to our study, which specifically targeted patients with anti-MDA5+DM-ILD, Lian et al’s [10] investigation encompassed a more expansive patient population, not restricting their analysis solely to anti-MDA5+DM-ILD cases. Furthermore, although their investigation incorporated HRCT findings for scoring purposes, our study deliberately excluded HRCT as a discriminating factor in the evaluation process, attributable to the recognized interobserver and intraobserver variability inherent in HRCT interpretation.

Ouyang et al [30] reported a novel matrix prediction model that includes baseline characteristics, such as fever, ferritin≥1250 µg/L, and positive carcinoma embryonic antigen, which can predict 6-month all-cause mortality in patients with anti-MDA5+DM-ILD. However, these prediction models lack thorough external validation due to their reliance on small and nonrepresentative populations (eg, individuals from a single hospital), primarily due to the rarity of the disease. Moreover, the HRCT imaging score and the radiomics-based prediction model [31,32] are overly complex and impractical for routine clinical use. In contrast, this study leveraged routinely collected data from real-world clinical practices in China to develop an optimal prediction model, which is now accessible as a web-based tool. This tool enables clinicians to calculate individualized risks of 3-month mortality based on easily obtainable clinical data, including RP-ILD, age, AST, CRP, NLR, LDH, ALB, and NLR. It is important to emphasize that in real-world clinical practice, such models should be used holistically, considering all key features, rather than relying on a single feature for risk prediction. Therefore, the models require the input of a comprehensive set of features to accurately assess the risk in patients with anti-MDA5+DM-ILD.

### Strengths and Limitations

Our study has several notable strengths. First, we conducted external validation in independent cohorts from 4 hospitals in China, encompassing various regions and settings. This inclusion of external cohorts 1 and 2 in China allows our findings to be representative of real-life clinical practice in our country. Second, we extracted, analyzed, and used data from routine clinical practice and finally established a practicable web-based algorithm for predicting 3-month mortality for patients with anti-MDA5+DM-ILD. Additionally, our study included a substantial number of patients with anti-MDA5+DM-ILD, further enhancing the robustness of our results.

However, it is important to acknowledge some limitations of our study. First, it was a retrospective study, which inherently introduces certain biases. Our patient population was sourced from large tertiary referral hospitals and consisted only of hospitalized patients, potentially leading to the inclusion of individuals with more severe disease. Second, although the predicted forced vital capacity (FVC<50%) was shown to independently predict 6-month all-cause mortality in patients with anti-MDA5-positive DM [29], we were unable to incorporate pulmonary function parameters into our prediction model due to missing data, particularly in patients with severe disease who had no opportunity to finish the pulmonary function test. Lastly, although the model demonstrated good discrimination and calibration in our study, the external validation cohort was limited to a Chinese population. It is necessary to develop a multiregion and multirace worldwide analysis and generate a more comprehensive and classical system to predict 3-month mortality for patients with anti-MDA5+DM-ILD.

### Conclusion

In summary, our use of ML techniques and routinely collected clinical features helped us successfully develop an LR model that reliably predicts the risk of 3-month mortality in patients with anti-MDA5+DM-ILD. To facilitate the translation of our findings into clinical practice, we created a web-based tool that allows clinicians to calculate individualized risks of 3-month mortality. This tool can guide risk-stratified approaches to patient care and treatment.

## Appendix

Multimedia Appendix 1 Performance metrics for 6 models and discrimination performance of death at different times.

Multimedia Appendix 2 Secondary outcomes for external cohort 1.

Multimedia Appendix 3 Secondary outcomes for external cohort 2.

## Abbreviations

anti-MDA5+DM-ILD: antimelanoma differentiation–associated gene 5 antibody–positive dermatomyositis–associated interstitial lung disease
ALB: albumin
ALT: alanine aminotransferase
ANA: antinuclear antibody
AST: aspartate aminotransferase
AUC: area under the receiver operating characteristic curve
CK: creatine kinase
CRP: C-reactive protein
CT: computed tomography
CTD: connective tissue disease
DCA: decision curve analysis
DM: dermatomyositis
EMR: electronic medical record
ESR: erythrocyte sedimentation rate
HRCT: high-resolution computed tomography
IIM: idiopathic inflammatory myopathies
IL: interleukin
ILD: interstitial lung disease
KL-6: Krebs von den Lungen-6
KNN: k-nearest neighbor
LDH: lactate dehydrogenase
LightGBM: Light Gradient Boosting Machine
LR: logistic regression
MDA5: melanoma differentiation–associated gene 5
ML: machine learning
MSA: myositis-specific antibody
NJDTH: Nanjing Drum Tower Hospital
NLR: neutrophil-to-lymphocyte ratio
PR: precision-recall
RF: rheumatoid factor
ROC: receiver operating characteristic
Ro52: Ro/SSA-52kDa antibody
RP-ILD: rapidly progressive interstitial lung disease
SHAP: Shapley Additive Explanations
SVM: support vector machine
WBC: white blood cell
XGBoost: Extreme Gradient Boosting

## Data Availability

Due to patient privacy, the data related to patients is not available for public access. However, it can be obtained from the corresponding author upon reasonable request, subject to approval by the institutional review board of the enrolled centers.
