# Correction: Mortality Risk Prediction in Patients With Antimelanoma Differentiation–Associated, Gene 5 Antibody–Positive, Dermatomyositis–Associated Interstitial Lung Disease: Algorithm Development and Validation

**DOI:** 10.2196/73983

**Published:** 2025-04-07

**Authors:** Hui Li, Ruyi Zou, Hongxia Xin, Ping He, Bin Xi, Yaqiong Tian, Qi Zhao, Xin Yan, Xiaohua Qiu, Yujuan Gao, Yin Liu, Min Cao, Bi Chen, Qian Han, Juan Chen, Guochun Wang, Hourong Cai

**Affiliations:** 1 Department of Respiratory and Critical Care Medicine, Nanjing Drum Tower Hospital, Affiliated Hospital of Medical School, Nanjing University Nanjing China; 2 Department of Pulmonary and Critical Care Medicine, Seventh Affiliated Hospital, Sun Yatsen University Shenzhen China; 3 Department of Pulmonary and Critical Care Medicine, General Hospital of Ningxia Medical University Yinchuan China; 4 Department of Respiratory and Critical Care Medicine, Third People’s Hospital of Chengdu Chengdu China; 5 Department of Respiratory and Critical Care Medicine, Affiliated Hospital of Xuzhou Medical University Xuzhou China; 6 National Center for Respiratory Medicine, First Affiliated Hospital of Guangzhou Medical University Guangzhou China; 7 China-Japan Friendship Hospital, Key Laboratory of Myositis (Beijing Key Laboratory for Immune Mediated Inflammatory Diseases), Department of Rheumatology Beijing China

In “Mortality Risk Prediction in Patients With Antimelanoma Differentiation–Associated, Gene 5 Antibody–Positive, Dermatomyositis–Associated Interstitial Lung Disease: Algorithm Development and Validation” (J Med Internet Res 2025;27:e62836) the authors made one change.

Affiliation 1 has been changed from:

Department of Respiratory and Critical Care Medicine, Affiliated Drum Tower Hospital, Nanjing University Medical School, Nanjing, China

to:

Department of Respiratory and Critical Care Medicine, Nanjing Drum Tower Hospital，Affiliated Hospital of Medical School，Nanjing University，Nanjing, China

The correction will appear in the online version of the paper on the JMIR Publications website, together with the publication of this correction notice. Because this was made after submission to PubMed, PubMed Central, and other full-text repositories, the corrected article has also been resubmitted to those repositories.

