# Supplementary figures and images for: Mortality Risk Prediction in Patients With Antimelanoma Differentiation–Associated, Gene 5 Antibody–Positive, Dermatomyositis–Associated Interstitial Lung Disease: Algorithm Development and Validation
Source: J Med Internet Res. 2025 Feb 5;27:e62836. doi: 10.2196/62836 (PMC11840371; doi:10.2196/62836)

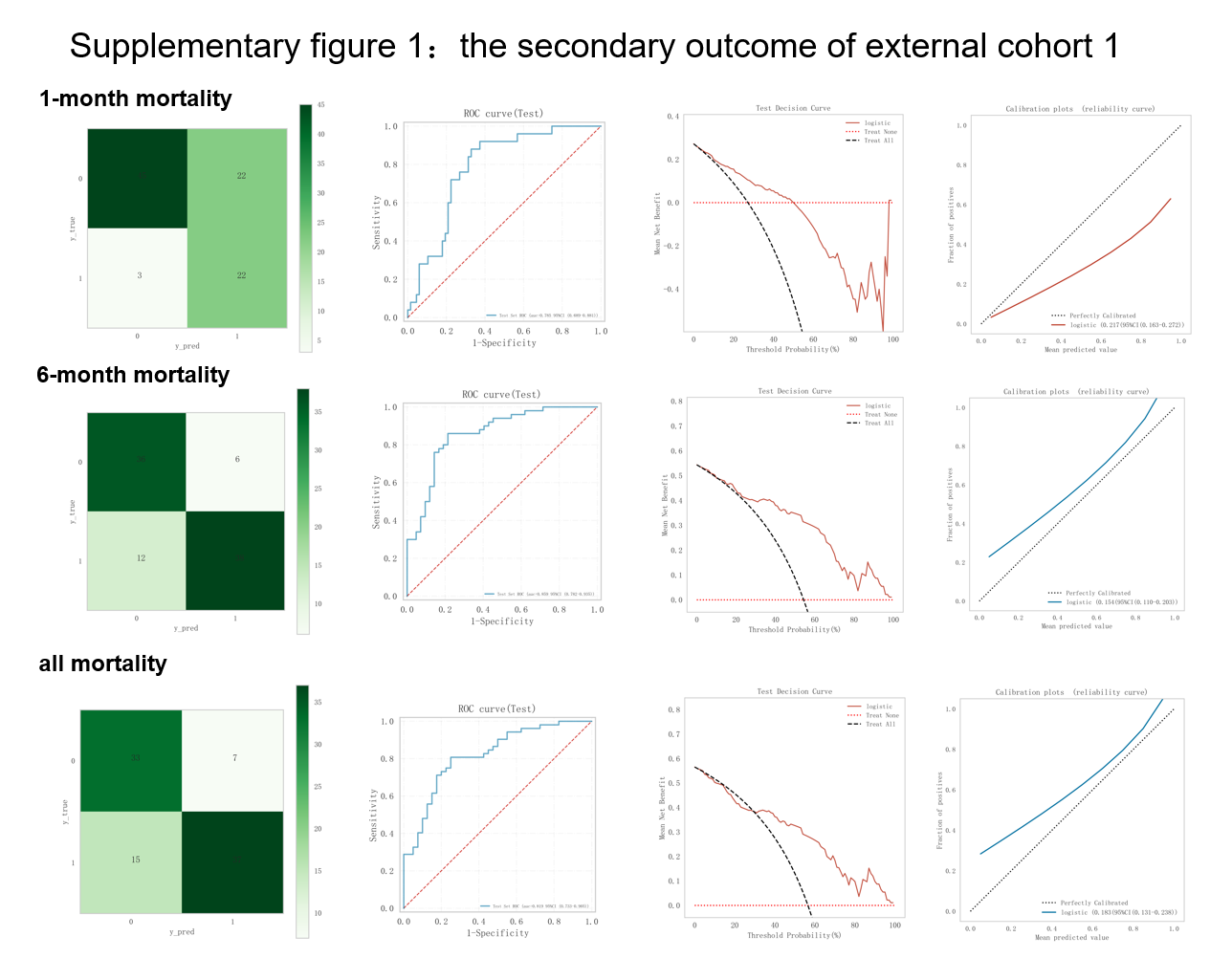

Supplement: Multimedia Appendix 2 [file jmir_v27i1e62836_app2.png]

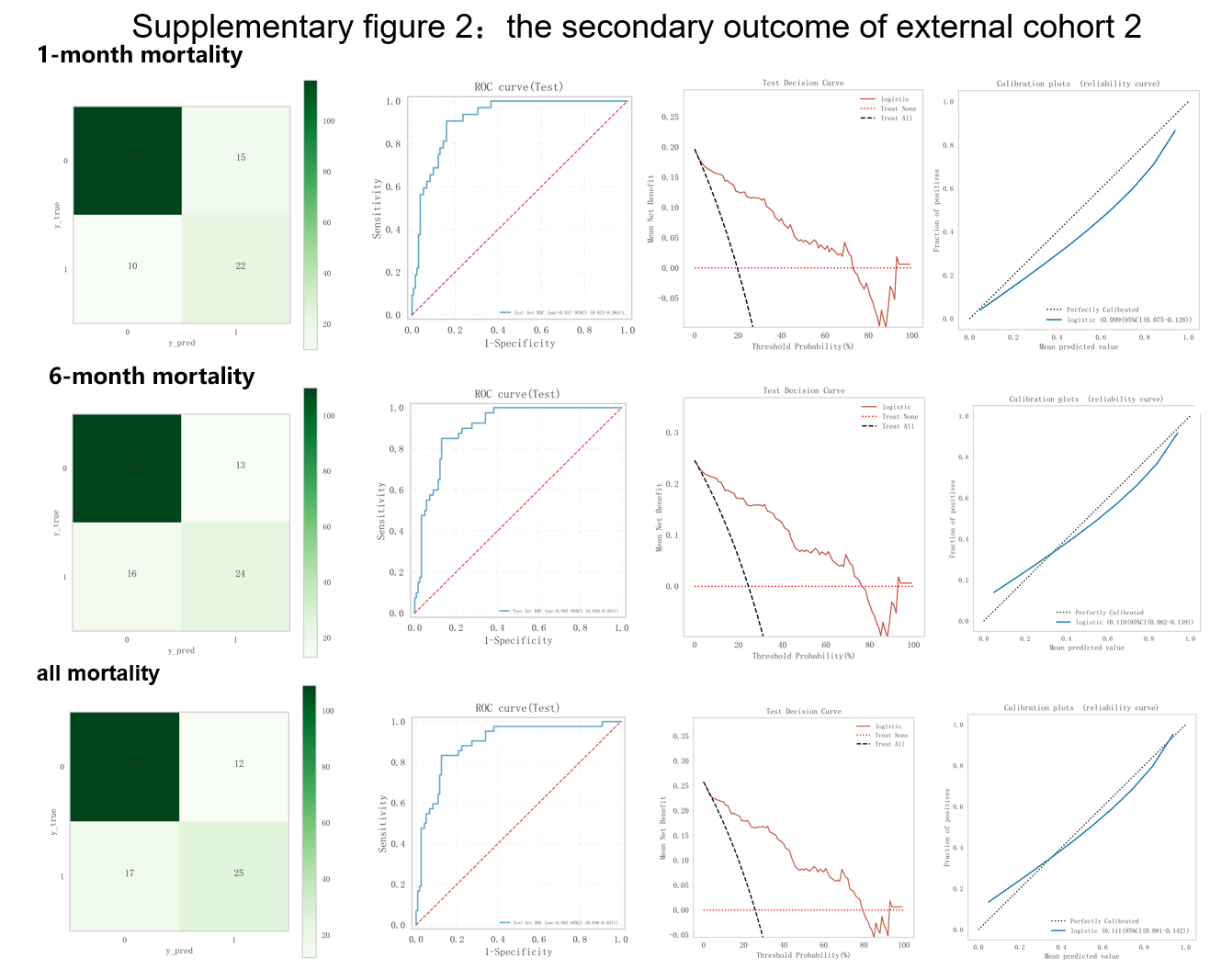

Supplement: Multimedia Appendix 3 [file jmir_v27i1e62836_app3.png]
